# Adaptation and Validation of Alternative Healthy Eating Index in Hemodialysis Patients (AHEI-HD) and Its Association with all-Cause Mortality: A Multi-Center Follow-Up Study

**DOI:** 10.3390/nu11061407

**Published:** 2019-06-21

**Authors:** Tuyen Van Duong, I-Hsin Tseng, Te-Chih Wong, Hsi-Hsien Chen, Tso-Hsiao Chen, Yung-Ho Hsu, Sheng-Jeng Peng, Ko-Lin Kuo, Hsiang-Chung Liu, En-Tzu Lin, Yi-Wei Feng, Shwu-Huey Yang

**Affiliations:** 1 School of Nutrition and Health Sciences, Taipei Medical University, Taipei 110, Taiwan; duongtuyenvna@gmail.com (T.V.D.); m507104010@tmu.edu.tw (I.-H.T.); b506101040@tmu.edu.tw (Y.-W.F.); 2 Department of Nutrition and Health Sciences, Chinese Culture University, Taipei 110, Taiwan; wdz5@ulive.pccu.edu.tw; 3 Division of Nephrology, Department of Internal Medicine, School of Medicine, College of Medicine, Taipei Medical University, Taipei 110, Taiwan; 570713@yahoo.com.tw (H.-H.C.); tsohsiao@tmu.edu.tw (T.-H.C.); yhhsu@tmu.edu.tw (Y.-H.H.); 4 Division of Nephrology, Department of Internal Medicine, Taipei Medical University Hospital, Taipei 110, Taiwan; 5 Department of Nephrology, Taipei Medical University-Wan Fang Hospital, Taipei 110, Taiwan; 6 Division of Nephrology, Department of Internal Medicine, Taipei Medical University- Shuang Ho Hospital, Taipei 110, Taiwan; 7 Division of Nephrology, Cathay General Hospital, Taipei 110, Taiwan; kennethpeng@cgh.org.tw; 8 Division of Nephrology, Taipei Tzu-Chi Hospital, New Taipei 231, Taiwan; kolinkuo8@gmail.com; 9 Department of Nephrology, Wei Gong Memorial Hospital, Miaoli 351, Taiwan; hc88liu@gmail.com; 10 Department of Nephrology, Lotung Poh-Ai Hospital, Yilan 265, Taiwan; nick.et.lin@gmail.com; 11 Research Center of Geriatric Nutrition, Taipei Medical University, Taipei 110, Taiwan; 12 Nutrition Research Center, Taipei Medical University Hospital, Taipei 110, Taiwan

**Keywords:** alternative healthy eating index, all-cause mortality, diet quality, diet quantity, hemodialysis, end-stage renal disease, principal component analysis, complex and multidimensional, validation, prospective cohort

## Abstract

A valid diet quality assessment scale has not been investigated in hemodialysis patients. We aimed to adapt and validate the alternative healthy eating index in hemodialysis patients (AHEI-HD), and investigate its associations with all-cause mortality. A prospective study was conducted on 370 hemodialysis patients from seven hospital-based dialysis centers. Dietary data (using three independent 24-hour dietary records), clinical and laboratory parameters were collected. The construct and criterion validity of original AHEI-2010 with 11 items and the AHEI-HD with 16 items were examined. Both scales showed reasonable item-scale correlations and satisfactory discriminant validity. The AHEI-HD demonstrated a weaker correlation with energy intake compared with AHEI-2010. Principle component analysis yielded the plateau scree plot line in AHEI-HD but not in AHEI-2010. In comparison with patients in lowest diet quality (tertile 1), those in highest diet quality (tertile 3) had significantly lower risk for death, with a hazard ratio (HR) and 95% confidence intervals (95%CI) of HR: 0.40; 95%CI: 0.18 – 0.90; *p* = 0.028, as measured by AHEI-2010, and HR: 0.37; 95%CI: 0.17–0.82; *p* = 0.014 as measured by AHEI-HD, respectively. In conclusion, AHEI-HD was shown to have greater advantages than AHEI-2010. AHEI-HD was suggested for assessments of diet quality in hemodialysis patients.

## 1. Introduction

End-stage renal disease (ESRD) patients treated with hemodialysis have been reported with high prevalence, and as being at high risk for cardiovascular disease and mortality [1,2,3]. This places significant financial burden upon the healthcare system [1]. Nutrition care has been reported as one of the strategical approaches to improve clinical outcomes, mortality, and healthcare expenditure [4]. Numbers of healthy diet guidelines have been developed to promote healthy eating behaviors and to prevent chronic diseases in the general population [5,6,7,8].

The healthy eating index (HEI), developed and updated in 2015, is a measure by which assess how well a set of foods aligns with recommendations of the Dietary Guidelines for Americans [9,10]. The alternative healthy eating index (AHEI) was created as an alternative to the HEI, based on foods and nutrients which can prevent chronic disease risks. The AHEI-2010 was updated and showed more advantages than the HEI in predicting major chronic disease and CVD risks [8,11]. Higher scores of dietary quality based on AHEI are strongly associated with lower risks of chronic diseases [8,12], cancer [13], and all-cause, cardiovascular, and cancer mortality [14,15,16,17,18]. Importantly, these eating indices were the basis for developing guidelines and educational materials in clinical practice.

In order to improve dialysis treatment and survival outcome, the diet and hemodialysis dyad approach is strongly recommended [19]. Better diet quality based on AHEI has demonstrated potential benefits for lower risk of mortality in chronic diseases. However, the AHEI has not been used to assess diet quality among hemodialysis patients. The current study aims to adapt and validate the AHEI-2010 and examine its association with all-cause mortality in hemodialysis patients after one-year minimum follow-up.

## 2. Materials and Methods

### 2.1. Study Design and Settings

A prospective cohort study was conducted in seven hospital-based hemodialysis centers. Two groups of patients were recruited at Taipei Medical University Hospital (from September to December 2013, and from November 2016 to January 2017). Patients were recruited from April to May 2014 at Taipei Medical University-Wan Fang Hospital, in December 2014 at Taipei Medical University-Shuang Ho Hospital, in March 2016 at Cathay General Hospital, in November 2016 at Taipei Tzu Chi General Hospital, from February to March 2017 at Wei Gong Memorial Hospital, and in April 2017 at Lotung Poh-Ai Hospital. Patients were followed-up for all-cause mortality up to April 2018.

### 2.2. Study Patients

**Figure 1:**
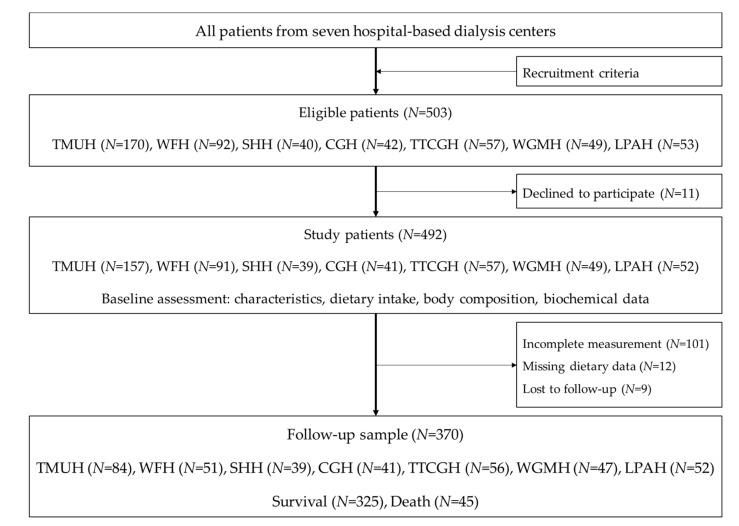
Sampling flow chart. TMUH, Taipei Medical University Hospital; WFH, Taipei Medical University-Wan Fang Hospital; SHH, Taipei Medical University-Shuang Ho Hospital; CGH, Cathay General Hospital; TTCGH, Taipei Tzu Chi General Hospital; WGMH, Wei Gong Memorial Hospital, and Lotung Poh-Ai Hospital. Recruitment criteria: aged 20 years and above, received thrice-weekly hemodialysis for at least 3 months, equilibrated Kt/V ≥ 1.2.

Patients recruited in the study were those aged 20 years and above, who had received three hemodialysis treatment per week for at least 3 months, and were equilibrated Kt/V ≥ 1.2. Patients excluded from the study were those diagnosed with one or more medical conditions including edema, pregnancy, amputation, hyperthyroidism, hypothyroidism, cancer or malignancy, as well as those who had received tube feeding, exhibited hepatic failure, been hospitalized less than one month prior to the recruitment, or who were scheduled for surgery. The recruitment criteria are described in [20]. A total sample of 370 patients were followed up and analyzed (Figure 1).

### 2.3. Assessments and Measurements

At baseline (September 2013 to April 2017), patient characteristics, dietary intake, body composition, and laboratory data were assessed. The information related to all-cause mortality was collected from patient medical records on the hospital electronic health record system.

### 2.4. Patients’ Characteristics 

All patient information was collected in the same week. Age, gender, dialysis vintage, Charlson comorbidity index, and body mass index were collected from medical charts. Physical activity level was assessed using the short version of the International Physical Activity Questionnaire [21]. 

### 2.5. Body Composition

Body composition was assessed by well-trained dietitians using the same bioelectrical impedance analysis device using multiple operating frequencies of 1, 5, 50, 250, 500, and 1000 kHz (InBody S10, Biospace, Seoul, Korea). Fat free mass (FFM), body fat mass (BFM) were analyzed in the current study.

### 2.6. Biochemical Parameters

Monthly fasting blood tests were performed in each selected hospital through automated and standardized methods. The laboratory test results were identical in different laboratories in selected hospitals. The studied biochemical parameters were total cholesterol (TC, mg/dL), triglyceride (TG, mg/dL), fasting blood glucose (FBG, mg/dL), hemoglobin (Hgb, g/dL), calcium (Ca, mg/dL), phosphorous (PO_4_, mg/dL), calcium and phosphate product (Ca × PO_4_), intact parathyroid hormone (iPTH, pg/mL), albumin (Alb, mg/dL), creatinine (Cr, mg/dL), and potassium (K, mEq/L). Other biochemical parameters were subsequently analyzed in the laboratory of Taipei Medical University Hospital, including values of low density lipoprotein cholesterol (LDL-C, mg/dL), high density lipoprotein cholesterol (HDL-C, mg/dL), homocysteine (Hcy, µmol/L) and high sensitivity C-reactive protein (hs-CRP, mg/dL). 

### 2.7. The Alternative Healthy Eating Index (AHEI) in Hemodialysis Patients

The dietary data were assessed using the 24-hour dietary recall form in paper format [22]. A qualified dietician instructed patients to complete the assessment forms on one dialysis day, one non-dialysis day, one non-dialysis day during the weekend, including meal time, meal location, food name, brand names, ingredients, portion or weight of food, and the cooking methods, oils used [20,23]. To confirm the data collected, dietitians used the 24-hour dietary recall form with common utensils to ask patients face-to-face or by phone call, as described previously [20,23,24,25]. If patients did not complete the form for one assessment, those with missing dietary intake data were not included in the analysis. Nutrients were analyzed using nutrition analysis software, e-Kitchen (Nutritionist edition, Enhancement plus 3, version 2009, Taichung, Taiwan), based on the Food Nutrition Database in Taiwan [26].

Index scores such as HEI and AHEI have been applied in different populations to investigate the association between dietary intake and health outcomes [8,10,12,14,15,16,18,24,27,28]. The AHEI was stronger in predicting major chronic diseases and CVD risks as compared to the original HEI [8,11]. The AHEI was used to examine the association between dietary intake and cardiovascular risks and event in type-2 diabetes patients in Taiwan. The AHEI index has shown its validity and applicability in Taiwan food culture [28]. Therefore, the AHEI-2010 was adapted and used in the current study.

In 2000, the NKF proposed that hemodialysis patients should avoid food containing high levels of phosphorus such as nuts, legumes, dairy products, and whole grains [29]. However, in 2016, the nutritional recommendations were modified; it was proposed that the restrictions on those items be eased, as they contain dietary fiber and healthy nutrients which may improve outcomes [30]. In addition, it was suggested that less restrictive dietary approaches be adopted to improve the autonomy and liberty of dialysis management [19]. Therefore, the intake of whole fruits and total vegetables was scored according to the Daily Food Guides in Taiwan [7], and the deciles of distribution of actual intake of whole grains, nuts and legumes, dairy products were used to obtain the index score. Lowe deciles of consumption of whole grains, nuts and legumes, and dairy products were suggested to obtain a maximum score.

Higher consumption of sugar-sweetened beverages are associated with higher metabolic syndrome [31] and mortality [32]. Therefore, no sugar-sweetened beverages and fruit juice was suggested [33], and a maximum score was obtained. 

The International Agency for Research on Cancer (IARC) announced that the consumption of processed meat is carcinogenic for humans (Group I) [34], and that it increases all-cause mortality [35,36]. However, the evidence on red meat consumption and the risk of cancer are not consistent; furthermore, red meat is rich in protein, vitamins and minerals, especially Heme-iron. Those nutrients are important for hemodialysis patients [29]. Therefore, red and/or processed meat in AHEI-2010 was divided into two items (red meat, and processed meat) in the current study. No consumption of processed meat, and consuming less than 1.5 servings a day of red meat were suggested, and the maximum score was obtained. 

The consumption of at least 1 serving of fish per week (especially spices rich in *n*-3 fatty acids eicosapentaenoic acid (EPA), and docosahexaenoic acid (DHA) such as salmon, sea bass, sardines, shrimp, trout, and mackerel) was suggested for improving cardiovascular outcomes [8,37], and inflammation in hemodialysis patients [38]. The maximum index score was achieved when the suggestions were met.

The EAT-Lancet commission recommends the consumption of unsaturated fats rather than saturated fats [33]. Therefore, unsaturated fatty acids (UFAs), rich oils (olive, soy, sunflower, tea, canola, rice, peanut, flax seed, and grape seed) are recommended. When the intake of oils meets the Daily Food Guides [7], the maximum score was achieved. The ‘UFAs rich oils’ item was used in the current study instead of the ‘PUFAs’ item in AHEI-2010. On the other hand, saturated fatty acids (SFAs) and rich oils (animal or palm oils) are not recommended in the current dietary guidelines. Therefore, patients consuming none of these will get the maximum score.

The Global Burden of Diseases, Injuries, and Risk Factors Study (GBD) 2016 Alcohol Collaborators suggested that zero alcohol consumption could minimize health risks [39]; under such circumstances, the maximum score was then obtained. The deciles of distribution of sodium intake were used to obtain the intake score [8]; the lowest decile was recommended to obtain the maximum score.

In addition, protein energy malnutrition is highly prevalent in hemodialysis patients. Restrictive dietary approaches are one of the causes [40]. Therefore, adequate energy and protein intake are critically important in securing the nutritional status of hemodialysis patients [23,29]. The total grain consumption and total protein intake were suggested based on the level of energy requirements according to the Daily Food Guides [7]. Furthermore, high biological value protein foods (soy products, meats [pork, beef, lamb, poultry, seafood/fish], and eggs) should account for at least 50% of total dietary protein [29]. Maximum index scores were obtained when this suggestion was followed.

The original AHEI-2010 items included whole fruits, vegetables, whole grains, sugar-sweetened beverages and fruit juice, nuts and legumes, fresh red meat, processed meat, fish, UFAs rich oils, alcohol and sodium. The modified version of AHEI for hemodialysis patients (AHEI-HD) included additional items such as total grains, total protein foods, high biological value (HBV) proteins, dairy products and SFAs rich oils. Most items were determined from the output of nutrition analysis software, while HBV proteins were calculated by summing the intake of soy products, meats (pork, beef, lamb, poultry, seafood/fish), and eggs. The maximum possible score of all items was 10. Intermediate intakes were scored proportionately between a minimum of 0 and a maximum of 10. Therefore, the sum scores ranged from 0 to 110 for the AHEI-2010 scale, and from 0 to 160 for the AHEI-HD scale.

### 2.8. Statistical Analysis

The continuous variables were checked for normality using a Shapiro-Wilk’s test (normal if *p* value > 0.05) [41], histograms, normal Q-Q plots and box plots. Descriptive analysis was used to describe the distributions of study variables via the mean and standard deviation for normally distributed variables, median and interquartile range for abnormally distributed variables, and frequency and percentage for categorical variables. The independent-samples *t*-test, Mann-Whitney U test, or Chi-square test were used to test the distributions of the study variables appropriately.

In order to evaluate the construct validity of AHEI-HD, a number of analyses were conducted. Firstly, Spearman correlation was used to estimate the correlation of eating index components, total scores and energy intake, which illustrated the performance of AHEI-2010 and AHEI-HD on assessing diet quality independent of diet quantity. The item-scale correlation was also assessed. Secondly, the principal component analysis (PCA) was used to examine the multidimensional characteristics of AHEI-2010 and AHEI-HD, for which the scree plot test was used. The scree plot is a subjective test, but it was commonly used in previous studies to determine the heterogeneity of the healthy eating index [10,27]. Thirdly, the known-group validity was conducted using *t*-tests to compare the distribution of AHEI-2010 and AHEI-HD scores between different age groups (<65 years vs. ≥ 65 years), gender (men vs. women), history of diabetes mellitus (DM vs. non-DM). 

In addition, criterion validity was assessed via hazard ratios for all-cause mortality by different tertile (categorical model) and by each tertile increment (continuous model) in AHEI-2010 and AHEI-HD index scores. The analysis was conducted using Cox proportional hazards models, as commonly used when examining the association between health eating index and mortality outcome [10,27]. Model 1 included eating index scores and all-cause mortality. In model 2, the confounders showed the association with all-cause mortality at *p* < 0.25 were selected [42]. The Spearman correlation among confounders was run to check and avoid co-linearity. If two or more variables moderately or strongly correlated with each other, one representative variable was kept in the multivariate model.

Data were analyzed by using the SPSS for Windows version 20.0 (IBM Corp., New York, USA). The significant level was set at *p*-value < 0.05.

### 2.9. Ethical Approvals

The study protocol was approved by the Ethics Committee of Taipei Medical University Joint Institutional Review Board (TMU-JIRB No. 201302024) for conducting in Taipei Medical University Hospital, Wan-Fang Hospital, Shuang Ho Hospital, and Wei-Gong Memorial Hospital; the ethical committee of Cathay General Hospital (CGH-OP104001) for conducting in Cathay General Hospital, and Lotung Poh-Ai Hospital; and the ethics committee of Taipei Tzu-Chi Hospital (04-M11-090). All patients signed the informed consent forms before the study conducted. 

## 3. Results 

In all, 45 deaths occurred over the median follow-up of 1.4 (1.0–3.2) years. Patients who died were those with older ages (*p* = 0.002), higher comorbidity indexes (*p* < 0.001), lower physical activity levels (*p* = 0.001), higher hs-CRP (*p* < 0.001), lower serum creatinine (*p* = 0.005), lower serum uric acid (*p* = 0.009), and lower eKt/V (*p* = 0.038; Table 1).

The total AHEI-2010, and AHEI-HD scores were 64.7 ± 13.4, and 98.7 ± 15.6, respectively; the scores were not significantly different between survivals and the deaths (Table 2).

Correlations between AHEI-HD components with total energy intake were mostly at low levels (*r* < 0.30), while fresh red meat (*r* = −0.33), sodium (*r* = −0.41), total grains (*r* = 0.60), total protein foods (*r* = 0.55) were at moderate levels. The AHEI-HD demonstrated a weaker correlation with energy intake (*r* = −0.05) as compared with AHEI-2010 (*r* = −0.19; Table 3).

The item-scale correlations ranged from low level, with *r* < 0.30 in total grains, HBV proteins, total protein foods, sodium, whole fruits, whole grains, alcohol, fresh red meat, to moderate level, with *r* ≥ 0.30 in total vegetables, fish, dairy products, nuts and legumes, processed meat, saturated fatty acids rich oils, unsaturated fatty acids rich oils, and sugar-sweetened beverages and fruit juice (Table 3).

**Figure 2:**
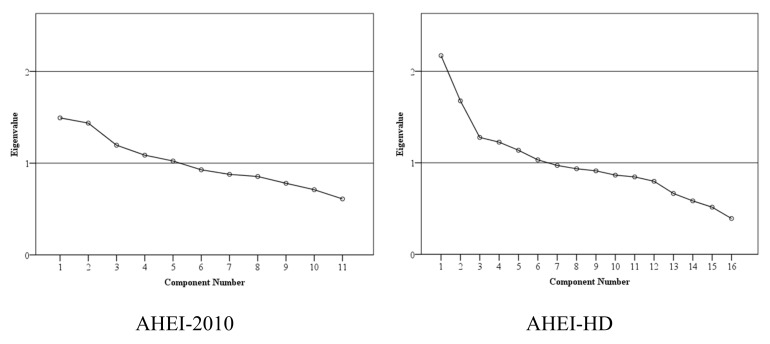
Scree plots from principle component analysis of alternative healthy eating index for hemodialysis patients. AHEI, alternative healthy eating index; HD, hemodialysis.

The results of principal component analysis (PCA) showed that AHEI-2010 consists of 5 dimensions as 5 components with eigenvalue > 1.0, and AHEI-HD consists of 6 dimensions as 6 components with eigenvalue > 1.0. The scree plot line appeared to plateau in AHEI-HD (Figure 2).

The AHEI-2010 score was significantly higher in aged ≥ 65 years and DM than that in women, aged < 65 years, and non-DM patients (*p* < 0.05). The AHEI-HD score was higher in patients aged ≥ 65 years, and DM than in patients aged < 65 years, and non-DM patients (*p* < 0.01; Table 4). 

The results of multivariate analysis showed that patients with the highest diet quality (tertile 3) had significantly lower risk of death than those in the lowest diet quality (tertile 1), with a hazard ratio (HR) and 95% confidence intervals (95% CI) of HR: 0.40; 95% CI: 0.18–0.90; *p* = 0.028 as measured by AHEI-2010, and HR: 0.37; 95% CI: 0.17–0.82; *p* = 0.014 as measured by AHEI-HD, respectively (Table 5). In addition, the hazards for all-cause mortality were significantly decreased for each tertile increment in AHEI-2010 score (HR: 0.63; 95% CI: 0.42–0.95; *p* = 0.027), and in AHEI-HD score (HR: 0.61; 95% CI: 0.41–0.91; *p* = 0.016), respectively (Table 5). In order to avoid the potential residual confounding possibility, confounders with *p* < 0.25 were adjusted in the multivariate analysis. The co-linearity among confounders was checked by running the Spearman correlation among selected variables with *p* < 0.25 from Supplementary Materials Table S1 (age, gender, hemodialysis vintage, CCI, physical activity, BMI, hs-CRP, FPG, TG, LDL-C, TC, serum phosphate, albumin, creatinine, uric acid, eKt/V). The results showed that creatinine moderately correlated with age, gender, albumin, and uric acid; while CCI strongly correlated with age; Kt/V moderately correlated with gender; BMI moderately correlated with TG; LDL-C strongly correlated with TC. Six confounders (age, gender, TG, TC, albumin, and uric acid) were excluded from the multivariate Cox hazard model. The final variables adjusted in multivariate Cox hazard model were hemodialysis vintage, CCI, physical activity, BMI, hs-CRP, FPG, LDL-C, serum phosphate, creatinine, and eKt/V (Table S2).

## 4. Discussion

In the current study, the alternative healthy eating index was adapted and validated to assess the diet quality in hemodialysis patients. The essential features of a healthy eating index were examined, including independence of quantity property, multidimensional characteristic, discriminant validity, and criterion validity. The clinical implication of the AHEI-HD is that patients should adhere to recommendations on all food items, rather than just focusing on a single item.

The construct validity of AHEI-2010 and AHEI-HD was illustrated by the low correlations between total scale, its components and total energy intake. These indicate that the diet quality assessment using AHEI-2010 or AHEI-HD were independent of diet quantity. It is an essential characteristic of a healthy eating index that the index score should not be dependent on the amount of foods eaten [10]. Moderate correlations between sodium, total grains and total protein foods were found in the current study. These could be explained by the fact that patients under hemodialysis should have a limited intake of sodium but high intake of total grains (especially refined grains) and total protein foods in order to provide adequate energy and protein to prevent protein energy wasting [29]. In addition, the total AHEI-HD score showed weaker correlation with total energy than AHEI-2010 score. This indicated that AHEI-HD is more independent of diet quantity in comparison with AHEI-2010 in hemodialysis patients.

The PCA illustrated the heterogeneity characteristic of AHEI-2010 and AHEI-HD. The PCA of AHEI-HD yielded the plateau scree plot and more components, which illustrated that AHEI-HD may have better performance in assessing multidimensional diet quality than AHEI-2010 in hemodialysis patients. The approach to evaluate the multidimensional feature of healthy index using PCA was also used in the previous studies [10,27].

In addition, the scores of AHEI-2010 and AHEI-HD were different between the groups (men and women, aged < 65 years and ≥ 65 years, non-DM and DM). The finding rebooted the construct validity of scales which reflected the variability across known groups who have differences in diet quality.

The item-scale correlations were low in the current study and previous study, as the internal consistency was not a necessary feature of healthy eating index [10,27]. This could be explained by the complexity and multidimensionality of diet quality [8,10].

Diet quality that scored highly on AHEI-2010 and AHEI-HD was significantly associated with 60% to 63% reduction for the hazard of all-cause mortality in hemodialysis patients. The findings are consistent with previous studies [10,17,18]. This provides evidence of criterion validity of the AHEI-2010 and AHEI-HD [10]. In the current analysis, the AHEI-HD demonstrated slightly better performance than AHEI-2010 in predicting mortality in hemodialysis patients.

The current study has some limitations. Firstly, the scores of the AHEI-2010 and AHEI-HD components were not sufficiently varied among individuals. This was elucidated in the high proportion of patient with highest index score in whole grains, sugar-sweetened beverages and fruit juice, nuts and legumes, fresh red meat, processed meat, fish, alcohol, sodium, HBV proteins, dairy product, and SFAs rich oils. On the one hand, this can be the result of a long period of hemodialysis treatment (5.7 ± 4.9 years) during which patients have received the nutritional education from doctors, nurses and dietitians. On the other hand, the average serum albumin and creatinine were 4.0 ± 0.4 mg/dL, and 11.1 ± 2.2 mg/dL; these values were higher than the cut-point of 3.5 mg/dL, and 7.5 mg/dL, respectively [43]. This partly explains the good nutritional status which might be one of the reflections of good eating behavior in our study population. Secondly, the measurement error might come from dietary recall. However, reliable laboratory data were used together with epidemiological methods that could promote the importance of diet quality assessments and nutritional care in hemodialysis patients. Finally, the recruited patients in our study were in stable conditions, in which the impact of dietary intake on mortality outcome was examined with less confounding effects. However, inclusion in the study may have biased the mortality risk from the total patient pool. Future studies are required to properly evaluate the AHEI-HD in a larger study population. 

## 5. Conclusions

In conclusion, the AHEI-HD (with 16 items) was found to have better performance than the AHEI-2010 (with 11 items) in hemodialysis patients. The AHEI-HD was a valid index to assess diet quality independent of diet quantity. The index has demonstrated a complex and multidimensional construct. It has also strongly predicted the mortality outcome that patients with the highest index score have a 60% to 63% lower risk of all-cause mortality compared with those with the lowest index score. However, the limitations of AHEI-HD need further validation in future studies.

## Figures and Tables

**Table 1:** Patient characteristics, body composition, biochemical parameters at baseline.

Variables	Total (*N* = 370)	Survival (*N* = 325)	Death (*N* = 45)	*p*-value ^1^
Age, years	60.7 ± 11.9	60.0 ± 11.8	66.0 ± 12.0	0.002
Gender, male	208 (56.2)	179 (55.1)	29 (64.4)	0.235
Hemodialysis vintage, year	5.7 ± 4.9	5.9 ± 5.1	4.4 ± 2.9	0.060
CCI	4.7 ± 1.6	4.6 ± 1.5	5.5 ± 1.6	<0.001
PA, MET-min/wk	4964.9 ± 1871.9	5082.6 ± 1872.4	4114.2 ± 1651.4	0.001
BMI, kg/m^2^	23.6 ± 3.9	23.6 ± 3.9	23.0 ± 3.5	0.348
Body composition				
FFM, kg	44.1 ± 11.1	44.0 ± 11.1	45.1 ± 9.1	0.507
BFM, kg	17.9 ± 8.3	18.1 ± 8.5	16.4 ± 7.4	0.212
Laboratory parameters				
hs-CRP, mg/dL	0.25 (0.11–0.60)	0.24 (0.10–0.52)	0.52 (0.16–1.00)	0.003
Hgb, g/dL	10.7 ± 1.1	10.8 ± 1.1	10.5 ± 1.3	0.174
FBG (mg/dL)	132.3 ± 58.4	131.9 ± 59.0	135.5 ± 54.9	0.697
Insulin, µU/mL	17.1 (8.8–31.7)	17.9 (9.0–32.3)	14.4 (7.2–28.0)	0.165
TG (mg/dL)	159.6 ± 111.9	162.8 ± 115.2	136.3 ± 81.5	0.137
HDL-C (mg/dL)	39.9 ± 22.0	39.3 ± 21.8	43.9 ± 23.1	0.204
LDL-C, mg/dL	100.3 ± 31.7	101.1 ± 32.1	94.8 ± 28.2	0.212
TC, mg/dL	165.6 ± 35.2	166.2 ± 35.0	160.9 ± 36.4	0.347
Ca, mg/dL	9.3 (8.9 – 9.7)	9.3 (8.7–9.7)	9.3 (9.0–9.7)	0.463
PO4, mg/dL	5.2 ± 1.2	5.2 ± 1.2	5.0 ± 1.3	0.381
iPTH, pg/mL	254.0 (95.9–450.8)	266.5 (103.9–451.3)	139.4 (53.0–407.0)	0.063
Hcy, µmol/L	20.7 ± 6.7	20.6 ± 6.6	21.2 ± 7.6	0.616
Albumin, g/dL	4.0 ± 0.4	4.0 ± 0.4	3.9 ± 0.4	0.229
Pre-BUN, mg/dL	72.8 ± 19.7	72.8 ± 20.2	73.4 ± 16.0	0.829
Creatinine, mg/dL	11.1 ± 2.2	11.2 ± 2.2	10.2 ± 1.5	0.005
K, mEq/L	4.8 (4.3–5.2)	4.8 (4.3–5.2)	4.8 (4.3–5.2)	0.876
Uric acid, mg/dL	7.3 ± 1.3	7.3 ± 1.3	6.8 ± 1.2	0.009
eKt/V	1.6 ± 0.3	1.6 ± 0.3	1.5 ± 0.2	0.038

CCI, Charlson comorbidity index; PA, physical activity; MET, metabolic equivalent minute/ week; BMI, body mass index; FFM, fat free mass; BFM, body fat mass; CRP, high-sensitivity C-reactive protein; Hgb, hemoglobin; FBG, fasting blood glucose; TG, triglyceride; HDL-C, high density lipoprotein cholesterol; LDL-C, low density lipoprotein cholesterol; TC, total cholesterol; Ca, serum calcium; PO4, serum phosphate; iPTH, intact parathyroid hormone; Hcy, homocysteine; Pre-BUN, pre-dialysis blood urea nitrogen; K, serum potassium; eKt/V, equilibrated Kt/V (dialysis adequacy).^1^ Data was presented as mean ± SD, median (interquartile range), percentage for normal distributed, non-normal distributed continuous variables, and categorical variables, appropriately. *p* values were calculated to compare the distribution of patients’ characteristics, body composition, and biochemical parameters between the survival and the death, using independent-samples T test, Mann-Whitney U test, or Chi-square test, appropriately.

**Table 2:** The scoring criteria and distribution of Alternative Healthy Eating Index in hemodialysis patients.

Component	Criteria		Actual Eating Index Distribution ^1^			
	Minimum Score of 0	Maximum Score of 10	Total (*N* = 370)	Survival (*N* = 325)	Death (*N* = 45)	*p*-value
Whole fruits, serving/d ^2^	0	≥2–4	1.7 (0.3–2.9)	1.8 (0.5–3.1)	0.7 (0.0–2.2)	0.007
Total vegetables, serving/d ^2^	0	≥3–5	3.3 (2.0–5.0)	3.3 (2.1–5.0)	3.1 (1.8–5.0)	0.590
Whole grains, serving/d ^3^	Highest decile	Lowest decile	361 (97.6)	318 (97.8)	43 (95.6)	0.350
Sugar-sweetened beverages and fruit juice, serving/d ^4^	>0	0	175 (47.3)	150 (46.2)	25 (55.6)	0.236
Nuts and legumes, serving/d ^3,5^	Highest decile	Lowest decile	269 (72.7)	236 (72.6)	33 (73.3)	0.919
Fresh red meat, serving/d ^3^	≥1.5	<1.5	154 (41.6)	139 (42.8)	15 (33.3)	0.229
Processed meat, serving/d ^6^	>0	0	160 (43.2)	134 (41.2)	26 (57.8)	0.036
Fish (EPA + DHA), serving/week	0	≥1	209 (56.5)	197 (60.6)	12 (26.7)	<0.001
UFAs rich foods, serving/d ^2,4,7^	0	≥4–8	3.5 (1.0–6.7)	3.5 (0.9–6.5)	3.4 (1.8–9.0)	0.247
Alcohol, drinks/d ^8^	>0	0	358 (96.8)	313 (96.3)	45 (100.0)	0.190
Sodium, mg/d ^5^	Highest decile	Lowest decile	217 (58.6)	193 (59.4)	24 (46.7)	0.440
Total AHEI-2010 score	0	110	64.7 ± 13.4	65.0 ± 13.3	62.5 ± 13.7	0.235
Total Grains, serving/d ^2^	0	≥8–18	5.3 ± 1.9	5.4 ± 1.9	5.0 ± 1.8	0.185
Total protein foods, serving/d ^2^	0	≥4.5–10	6.0 ± 2.3	6.0 ± 2.3	5.8 ± 2.5	0.636
HBV proteins, % total protein ^3^	0	≥50	360 (97.3)	318 (97.8)	42 (93.3)	0.080
Dairy products, serving/d ^3^	Highest decile	Lowest decile	268 (72.4)	237 (72.9)	31(68.9)	0.570
SFAs rich foods, serving/d ^4,7^	>0	0	195 (52.7)	158 (48.6)	37 (82.2)	<0.001
Total AHEI-HD score	0	160	98.7 ± 15.6	98.7 ± 15.6	98.7 ± 15.3	0.992

EPA, eicosapentaenoic acid; DHA, docosahexaenoic acid; UFA, unsaturated fatty acids; AHEI, alternative healthy eating index; HBV, high biological value; SFA, saturated fatty acids; HD, hemodialysis. ^1^ The actual index distributions were presented as mean ± standard deviation, median and interquartile range, or proportion of maximum score, appropriately. *p* values were calculated to compare the distribution of alternative health eating index and its components between the survival and the death, using independent-samples *t*-test, Mann-Whitney U test, or Chi-square test, appropriately. ^2^ Recommended amounts of food groups expressed per energy requirement level, found in the Daily Food Guide in Taiwan. ^3^ Suggested by the Kidney Disease Outcomes Quality Initiative (K/DOQI) Clinical Practice Guidelines for Nutrition in Chronic Renal Failure. ^4^ Suggested by the EAT–Lancet Commission. ^5^ The deciles of distribution of actual intake in the hemodialysis patients were used to obtain the intake score. ^6^ Suggested by the World Health Organization (WHO)’s International Agency for Research on Cancer (IARC). ^7^ UFAs rich oils consisting all vegetable oils (olive, soy, sunflower, tea, canola, rice, peanut, Flax seed, and Grape seed); SFAs rich oils consisting animal and palm oils. ^8^ Suggested by the Global Burden of Diseases (GBD) 2016 Alcohol Collaborators.

**Table 3:** Correlations of AHEI-2010 and AHEI-HD components and total scores with total energy intake (*N* = 370).

	Whole Fruits	Total Vegetables	Whole Grains	SSB and Fruit Juice	Nuts and Legumes	Fresh Red Meat	Processed Meat	Fish (EPA + DHA)	UFAs Rich Oils	Alcohol	Sodium	Total Grains	Total Protein Foods	HBV Proteins	Dairy Products	SFAs Rich Oils	AHEI-2010	AHEI-HD
Total vegetables	0.11																	
Whole grains	−0.01	−0.01																
SSB and fruit juice	−0.06	0.14	0.08															
Nuts and legumes	−0.07	0.01	0.08	0.28														
Fresh red meat	−0.08	−0.09	−0.01	0.05	0.01													
Processed meat	0.06	0.16	−0.04	0.06	0.08	0.08												
Fish (EPA + DHA)	0.12	0.08	0.01	−0.09	−0.07	0.01	−0.09											
UFAs rich oils	0.06	0.23	0.01	0.09	−0.03	−0.07	0.07	0.07										
Alcohol	−0.06	−0.04	−0.03	0.05	0.09	0.12	0.04	0.00	0.06									
Sodium	−0.01	−0.13	0.01	0.03	−0.01	0.11	0.10	0.10	−0.11	0.08								
Total Grains	0.00	0.13	0.01	−0.07	−0.03	−0.19	−0.11	0.01	0.07	0.00	−0.27							
Total protein foods	0.06	0.21	0.03	−0.15	−0.05	−0.46	−0.19	0.23	0.19	−0.08	−0.29	0.27						
HBV proteins	0.09	0.10	−0.03	−0.08	−0.07	−0.20	0.04	0.06	0.11	−0.03	0.01	0.09	0.05					
Dairy products	−0.03	−0.03	0.05	0.14	0.04	0.05	0.06	0.07	−0.02	0.05	0.01	−0.05	−0.06	−0.06				
SFAs rich oils	−0.07	−0.07	−0.01	0.10	0.08	0.05	0.12	−0.14	0.37	0.10	−0.07	−0.13	−0.12	−0.06	0.07			
AHEI-2010	0.14	0.33	0.12	0.52	0.42	0.41	0.47	0.33	0.32	0.23	0.20	−0.12	−0.17	−0.04	0.14	0.14		
AHEI-HD	0.11	0.30	0.13	0.48	0.38	0.28	0.41	0.30	0.44	0.22	0.07	−0.01	−0.03	−0.02	0.37	0.42	0.89	
Total energy intake	0.05	0.20	0.02	−0.13	−0.13	−0.33	−0.12	0.06	0.27	−0.15	−0.41	0.60	0.55	0.08	−0.07	−0.07	−0.19	−0.05

SSB, Sugar-sweetened beverages; EPA, eicosapentaenoic acid; DHA, docosahexaenoic acid; UFA, unsaturated fatty acids; HBV, high biological value; SFA, saturated fatty acids; AHEI, alternative healthy eating index; HD, hemodialysis.

**Table 4:** Distributions of AHEI-2010 and AHEI-HD scores in different known groups of gender, age, and history of diabetes mellitus (*N* = 370).

		AHEI-2010	AHEI-HD
	*N*	Mean ± SD	Mean ± SD
Gender			
Women	162	66.4 ± 13.3	99.3 ± 14.7
Men	208	63.4 ± 13.3	98.2 ± 16.3
*p* value		0.031	0.492
Age			
< 65 years	239	63.3 ± 13.4	97.0 ± 15.9
≥ 65 years	131	67.3 ± 13.0	101.8 ± 14.5
*p* value		0.006	0.004
DM history			
Non-DM	223	63.0 ± 12.6	96.7 ± 14.6
DM	147	67.3 ± 14.1	101.8 ± 16.5
*p* value		0.003	0.002

AHEI, alternative healthy eating index; HD, hemodialysis; SD, standard deviation; DM, diabetes mellitus. *p* values calculated using independent-samples *t*-test.

**Table 5:** Hazard ratios for all-cause mortality according to the tertile of alternative healthy eating index scores via Cox proportional hazards model (*N* = 370).

	Death	Model 1		Model 2	
	(*N* = 45)	HR (95% CI)	*p*	HR (95% CI)	*p*
Categorical model					
Total AHEI-2010 score					
Tertile 1 (27.4–57.5)	16	Reference		Reference	
Tertile 2 (57.5–71.1)	18	0.97 (0.49–1.90)	0.926	0.64 (0.31–1.34)	0.237
Tertile 3 (71.1–95.9)	11	0.67 (0.31–1.45)	0.308	0.40 (0.18–0.90)	0.028
Total AHEI-HD score					
Tertile 1 (64.1–91.6)	15	Reference		Reference	
Tertile 2 (91.6–106.6)	16	0.83 (0.41–1.70)	0.616	0.57 (0.26–1.25)	0.161
Tertile 3 (106.6–135.0)	14	0.71 (0.34–1.48)	0.363	0.37 (0.17–0.82)	0.014
Continuous model					
Per each tertile increment in AHEI-2010 score	45	0.83 (0.57–1.20)	0.318	0.63 (0.42–0.95)	0.027
Per each tertile increment in AHEI-HD score	45	0.84 (0.58–1.22)	0.363	0.61 (0.41–0.91)	0.016

HR, hazard ratio; CI, conference interval; AHEI, alternative healthy eating index; HD, hemodialysis. Model 1: Univariate analysis between eating index scores and all-cause mortality. Model 2: Adjusted for hemodialysis vintage, and Charlson comorbidity index, physical activity level, body fat mass, high sensitivity C-reactive protein, fasting plasma glucose, low density lipoprotein cholesterol, serum phosphate, creatinine, dialysis adequacy (eKt/V).

## Supplementary Materials

The following are available online at https://www.mdpi.com/2072-6643/11/6/1407/s1, Table S1: Associations of patients’ characteristics, body composition, laboratory parameters with all-cause mortality via univariate Cox proportional hazards model (*N* = 370), Table S2: Spearman correlations among selected confounders (*N* = 370).
