# Supplementary material for: Adaptation and Validation of Alternative Healthy Eating Index in Hemodialysis Patients (AHEI-HD) and Its Association with all-Cause Mortality: A Multi-Center Follow-Up Study
Source: Nutrients. 2019 Jun 21;11(6):1407. doi: 10.3390/nu11061407 (PMC6627491; doi:10.3390/nu11061407)
Supplement: Supplementary file 1 [file nutrients-11-01407-s001.zip › AHEI and Mortality-Table S2-2019.05.30-R.docx]

**Table S2:** Spearman correlations among selected confounders (*N*=370).

|  | Age | Gender | HD vintage, year | CCI | PA, MET-min/wk | BFM, kg | hs-CRP, mg/dL | FPG, mg/dL | TG, mg/dL | LDL, mg/dL | TC, mg/dL | PO4, mg/dL | Albumin, g/dL | Creatinine, mg/dL | Uric acid, mg/dL |
| --- | --- | --- | --- | --- | --- | --- | --- | --- | --- | --- | --- | --- | --- | --- | --- |
| Gender | 0.023 |  |  |  |  |  |  |  |  |  |  |  |  |  |  |
| HD vintage, year | -0.136 | -0.106 |  |  |  |  |  |  |  |  |  |  |  |  |  |
| CCI | 0.750 | 0.041 | -0.188 |  |  |  |  |  |  |  |  |  |  |  |  |
| PA, MET-min/wk | 0.019 | 0.080 | 0.040 | 0.001 |  |  |  |  |  |  |  |  |  |  |  |
| BFM, kg | 0.056 | -0.117 | -0.145 | 0.132 | 0.000 |  |  |  |  |  |  |  |  |  |  |
| hs-CRP, mg/dL | 0.103 | 0.113 | -0.058 | 0.091 | -0.030 | 0.209 |  |  |  |  |  |  |  |  |  |
| FPG, mg/dL | 0.218 | 0.025 | -0.262 | 0.249 | 0.113 | 0.241 | 0.152 |  |  |  |  |  |  |  |  |
| TG, mg/dL | -0.065 | 0.003 | -0.142 | 0.038 | -0.013 | 0.396 | 0.218 | 0.293 |  |  |  |  |  |  |  |
| LDL, mg/dL | -0.067 | -0.141 | -0.027 | -0.106 | -0.042 | 0.178 | -0.045 | -0.085 | 0.098 |  |  |  |  |  |  |
| TC, mg/dL | -0.049 | -0.249 | -0.032 | -0.111 | -0.083 | 0.121 | -0.092 | -0.076 | 0.159 | 0.793 |  |  |  |  |  |
| PO4, mg/dL | -0.263 | 0.088 | 0.010 | -0.203 | -0.085 | 0.019 | 0.057 | -0.182 | 0.038 | 0.069 | 0.027 |  |  |  |  |
| Albumin, g/dL | -0.213 | 0.108 | 0.027 | -0.176 | -0.031 | -0.108 | -0.144 | -0.297 | 0.013 | 0.106 | 0.128 | 0.139 |  |  |  |
| Creatinine, mg/dL | -0.339 | 0.374 | 0.125 | -0.267 | 0.079 | 0.002 | -0.105 | -0.210 | 0.069 | 0.083 | -0.025 | 0.275 | 0.319 |  |  |
| Uric acid, mg/dL | -0.184 | -0.060 | 0.062 | -0.165 | -0.014 | 0.142 | 0.117 | -0.050 | 0.183 | 0.052 | 0.029 | 0.215 | 0.015 | 0.334 |  |
| eKt/V | 0.096 | -0.405 | 0.281 | 0.033 | 0.023 | -0.180 | -0.202 | -0.104 | -0.201 | 0.109 | 0.137 | -0.187 | -0.126 | -0.253 | -0.003 |

HD, hemodialysis; CCI, Charlson comorbidity index; PA, physical activity; MET, metabolic equivalent minute/ week; BFM, body fat mass; hs-CRP, high-sensitivity C-reactive protein; FPG, fasting plasma glucose; TG, triglyceride; LDL-C, low density lipoprotein cholesterol; TC, total cholesterol; PO4, serum phosphate; eKt/V, equilibrated Kt/V (dialysis adequacy).
