# Supplementary material for: Adaptation and Validation of Alternative Healthy Eating Index in Hemodialysis Patients (AHEI-HD) and Its Association with all-Cause Mortality: A Multi-Center Follow-Up Study
Source: Nutrients. 2019 Jun 21;11(6):1407. doi: 10.3390/nu11061407 (PMC6627491; doi:10.3390/nu11061407)
Supplement: Supplementary file 1 [file nutrients-11-01407-s001.zip › AHEI and Mortality-Table S1-2019.05.30.docx]

**Table S1:** Associations of patients’ characteristics, body composition, laboratory parameters with all-cause mortality via univariate Cox proportional hazards model (N=370).

| Variables | HR (95%CI) | *p-*value |
| --- | --- | --- |
| Age, year | 1.05 (1.02 - 1.07) | 0.001 |
| Gender, male | 1.78 (0.97 - 3.30) | 0.065 |
| Hemodialysis vintage, year | 0.93 (0.86 - 1.00) | 0.055 |
| CCI | 1.35 (1.11 - 1.64) | 0.003 |
| PA, MET-min/wk | 0.93 (0.84 - 1.04) | 0.218 |
| BMI, kg/m^2^ | 0.96 (0.89 - 1.04) | 0.330 |
| Body composition |  |  |
| FFM, kg | 1.01 (0.99 - 1.04) | 0.296 |
| BFM, kg | 0.97 (0.94 - 1.01) | 0.179 |
| Laboratory parameters |  |  |
| hs-CRP, mg/dL | 1.24 (1.08 - 1.42) | 0.002 |
| Hgb, g/dL | 0.88 (0.68 - 1.13) | 0.310 |
| FPG (mg/dL) | 1.00 (1.00 - 1.01) | 0.218 |
| Insulin, µU/mL | 1.00 (0.99 - 1.01) | 0.935 |
| TG (mg/dL) | 1.00 (0.99 - 1.00) | 0.212 |
| HDL-C (mg/dL) | 1.01 (0.99 - 1.02) | 0.396 |
| LDL-C, mg/dL | 0.99 (0.98 - 1.00) | 0.089 |
| TC, mg/dL | 0.99 (0.99 - 1.00) | 0.181 |
| Ca, mg/dL | 0.99 (0.90 - 1.09) | 0.844 |
| PO4, mg/dL | 0.83 (0.64 - 1.09) | 0.179 |
| iPTH, pg/mL | 1.00 (0.99 - 1.00) | 0.251 |
| Hcy, µmol/L | 0.99 (0.95 - 1.04) | 0.779 |
| Albumin, g/dL | 0.43 (0.21 - 0.88) | 0.021 |
| Pre-BUN, mg/dL | 0.99 (0.98 - 1.01) | 0.281 |
| Creatinine, mg/dL | 0.79 (0.68 - 0.91) | 0.002 |
| K, mEq/L | 0.98 (0.79 - 1.21) | 0.817 |
| Uric acid, mg/dL | 0.73 (0.60 - 0.89) | 0.002 |
| eKt/V | 0.19 (0.06 - 0.65) | 0.008 |

HR, hazard ratio; CI, conference interval; CCI, Charlson comorbidity index; PA, physical activity; MET, metabolic equivalent minute/ week; BMI, body mass index; FFM, fat free mass; BFM, body fat mass; CRP, high-sensitivity C-reactive protein; Hgb, hemoglobin; FPG, fasting plasma glucose; TG, triglyceride; HDL-C, high density lipoprotein cholesterol; LDL-C, low density lipoprotein cholesterol; TC, total cholesterol; Ca, serum calcium; PO4, serum phosphate; iPTH, intact parathyroid hormone; Hcy, homocysteine; Pre-BUN, pre-dialysis blood urea nitrogen; K, serum potassium; eKt/V, equilibrated Kt/V (dialysis adequacy).
